# Unsuccessful Intubation and Stabilization by Laryngeal Mask Airway in the Delivery Room: A Case of Tracheal Atresia

**DOI:** 10.1155/2021/9983153

**Published:** 2021-08-26

**Authors:** Mark Cody Smith, Autumn Kiefer, C. Eric Bailey

**Affiliations:** ^1^Department of Pediatrics, West Virginia University, Morgantown, WV, USA; ^2^Department of Otolaryngology, West Virginia University, Morgantown, WV, USA

## Abstract

A term male newborn infant was apneic at birth, and endotracheal intubation was unsuccessful. He was stabilized for transport with a laryngeal mask airway. Laryngoscopy revealed tracheal atresia with intrathoracic distal tracheoesophageal fistula. A laryngeal mask airway may provide ventilation in tracheal atresia patients with a fistula.

## 1. Introduction

Over 50% of congenital anomalies are detected by prenatal ultrasound, with improved rates in those with multiple anomalies or high-risk mothers [1, 2]. The sensitivity for lethal anomalies is up to 89% [3], and prenatal detection can allow for life-saving delivery room interventions. For example, tracheal compression by a neck mass historically carried a very high mortality, but a scheduled extrautero intrapartum treatment (EXIT) procedure with emergent tracheostomy can greatly alter prognosis [4]. However, some airway anomalies are not evident until birth, or in severe cases, until autopsy [5]. Despite appropriate prenatal care, major airway anomalies can impede routine airway management in the delivery room [6]. One of the most severe anomalies is tracheal atresia.

Tracheal atresia (TA) is a rare congenital airway defect with an estimated incidence of less than 1 per 50,000 with a 2 : 1 male predominance [7]. This lethal anomaly, originally described in 1900, is characterized by the complete absence or interruption of the trachea and is rarely reported in the literature [8]. Faro's classification system is the most comprehensive and categorizes TA into 7 types, A through G, with type B being the most common (46%), followed by types B (31%) and then E (15%) [9]. Other concomitant malformations are often present, most commonly involving the gastrointestinal system. TA has been associated with VACTERL syndrome, TACRD, Fraser syndrome, CHAOS syndrome, and Turner syndrome [10]. Most of these infants are not diagnosed prenatally and therefore present in the delivery room with severe respiratory distress, cyanosis, no audible cry, and inability to pass the endotracheal tube. Airway management during resuscitation has been accomplished either with mask ventilation as well as unintentional or intentional esophageal intubation. There are only two cases in the literature that were managed via the EXIT procedure in which the infants were diagnosed with CHAOS syndrome prenatally, so TA was known [10]. In this case presentation, we describe the first successful use of laryngeal mask airway (LMA) in the delivery room in management of an infant with TA.

## 2. Case Presentation

A 3.46 kg, 38 weeks gestation male infant was born at an outside hospital via spontaneous vaginal delivery following an uncomplicated pregnancy with normal amniotic fluid index. He had no spontaneous cry and heart rate was <60 bpm. Despite tactile stimulation, he remained apneic and cyanotic. With bag-mask ventilation, his heart rate increased to 100 bpm, but he remained apneic. Endotracheal intubation was attempted multiple times without success. Our tertiary care center was contacted for transfer at one hour of life due to persistent apnea with SpO_2_ between 70 and 80%. It was initially unclear if provider experience was a factor in the intubation's difficulty.

Our pediatric transport team arrived and found the patient to be mottled, cyanotic, and without spontaneous movement or respiration. He was receiving bag-mask ventilation and frequent gastric decompression due to significant abdominal distension. The transport team attempted intubation with 3.5 mm and 2.5 mm endotracheal tubes (ETT). Neither ETT would pass beyond about 1 cm through the vocal cords. A size 1 laryngeal LMA was placed, and the patient's SpO_2_ increased to 80–90%. Pulses remained >100 bpm, and bilateral breath sounds were present. Arterial blood gas (ABG) showed a pH < 6.8, PCO_2_ > 140 mmHg, and an incalculable base deficit. Saline boluses and sodium bicarbonate were given for hypoperfusion and marked acidosis. The patient was transported to our institution with a LMA without further decompensation.

**Figure 1:**
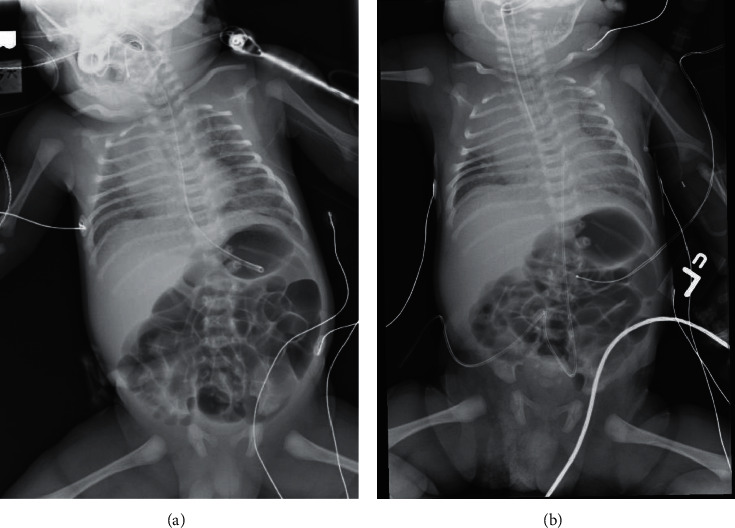
Radiograph of the chest and abdomen on admission. (a) The lungs are aerated bilaterally with LMA in place and show hazy parenchymal opacities bilaterally. There is a hemivertebra at T12 and 11 rib pairs on the right. (b) Similar radiograph with the esophageal tube in place in addition to removal of the nasogastric tube and placement of umbilical catheters.

On arrival to our institution, diminished musical breath sounds were heard bilaterally, with high pitched stridor and wheezing. The patient's heart rate was 160 bpm, and SpO_2_ was 76% with 100% oxygen via LMA. We considered tracheal stenosis, tracheal web, or compression of the airway by a mass, and thus emergently consulted otolaryngologist and anesthesia. Admission radiograph showed the lungs to be well aerated bilaterally via LMA with hazy opacities, hemivertebra at T12, and 11 rib pairs on right (Figure 1). A 3/6 harsh systolic murmur was present. No dysmorphic features were seen on cursory exam, but he was bleeding from the nares. The abdomen was very distended, so gastric suction was resumed which caused immediate bradycardia. Endotracheal intubation was attempted by both the neonatologist and anesthesiologist without success. A thick, pink tissue plane was noted about 1 cm past the patient's normal appearing vocal cords. The tissue did not rupture with gentle pressure of the ETT. There was no cough, gag response, or bleeding from the airway. LMA was placed again, and his heart rate and SpO_2_ once again stabilized. Repeat ABG revealed pH 6.8, CO_2_ 140 mmHg, O_2_ 62 mmHg, an incalculable base deficit, and lactate 8.9 mmol/L. Extremities were held in extension without spontaneous movement. Otolaryngology took the patient for rigid laryngoscopy and tracheostomy. Fresh frozen plasma was infused due to bleeding with an INR of 2.09. Our team prepared to initiate therapeutic hypothermia for severe hypoxic ischemic encephalopathy (HIE) if an airway could be surgically established.

**Figure 2:**
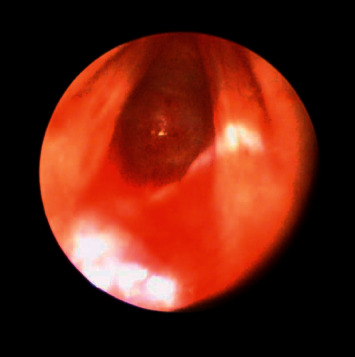
Rigid laryngoscopy of vocal cords and subglottis. The petechiae present on the distal wall of the blind-ended laryngeal pouch from previous intubation attempts are noted. No tracheal rings are present.

Rigid laryngoscopy showed a blind tracheal pouch just beyond the vocal cords (Figure 2; Supplementary Video (available here)). As air did enter the lungs, esophagoscopy was performed and located a “pinhole” distal trachealesophageal fistula (TEF). Stomach decompression reduced airflow through the TEF. A tracheostomy could not be performed as the distal trachea could not be accessed suprasternally.

Risks and benefits of transfer for cardiothoracic surgery intervention were discussed with the closest referral center and his parents. A repeat ABG at four hours of life showed pH 6.81, PCO_2_ 104 mmHg, PO_2_ 62 mmHg, base deficit 20 mmol/L, and lactate 8.1 mmol/L. Amplitude EEG showed burst suppression, and the patient had clinical seizures. Hypoxic ischemic injury to the liver and heart was evidenced by coagulopathy, elevated troponin-I (167), and elevated creatine kinase (1 093 units/L). Intentional esophageal intubation was performed with a 4.5 mm tube, and the depth was adjusted to optimize SpO_2_ and ventilation through the TEF. SpO_2_ reached 100%, but he still had periodic cardiorespiratory decompensations. Given the exceeding high mortality of TA and the ongoing evidence of severe HIE, we transitioned to comfort care and the baby died at about 7 hours of life. Postmortem exam revealed bilateral 2, 3 syndactylies of the toes and a deep sacral dimple. Parents declined autopsy, but genetic testing including karyotype (46, *XY*), comparative genomic hybridization, and Smith–Lemli–Opitz screen were all normal. Given his major and minor anomalies, VACTERL was suspected.

## 3. Discussion

TA with distal TEF is a rare and almost uniform lethal congenital anomaly. The incidence is estimated at 0.002% of children's hospital admissions [11], with a 2 : 1 male predominance [12]. The most common type of TA involves fistula formation between the esophagus and carina, with a blind laryngeal pouch. Prenatal diagnosis of TA is unusual but has been described in cases of complete atresia with fetal hydrops and increased lung echogenicity [13, 14]. When a fistula is present, the infant may still develop polyhydramnios, but our patient did not have any prenatal indicators.

In the delivery room, tracheal atresia should be considered in neonates with apnea, absence of crying, and inability to intubate, particularly by skilled providers [15]. A respiratory decompensation triggered by gastric decompression should also raise the suspicion that ventilation may be occurring by an atypical route. Removing excess air from the stomach typically improves ventilation by decreasing pressure on the diaphragm; however, in patients with TA with TEF, gastric decompression can decrease esophageal pressure which decreases flow through a high resistance TEF. Gastric decompression may have triggered periods of bradycardia in our patient. Reflux of gastric secretions or saliva can block airflow through a tiny TEF and predispose to pneumonia, suggesting some long-term benefits of decompression [5].

We also describe the first successful use of a LMA in a patient with TA with TEF. Since prenatal diagnosis is rare, these patients are not necessarily born at tertiary care centers. Stabilization with a LMA may be preferable to intentional esophageal intubation when the diagnosis is still in question since many types of difficult airways can benefit from LMA placement. Bercker described failure of a LMA in a 32 weeks' gestation, a 1500 g neonate with TA with high TEF [6]. Our patient may have responded better due to his larger size or lower position of the TEF. Esophageal intubation still offers the possibility of prolonged ventilation. One child survived 6 weeks with esophageal intubation before dying of pneumonia [16], and another lived 6 years before dying of esophageal hemorrhage [17].

Since tracheostomies are usually unsuccessful in TA, this anomaly will likely remain fatal until a feasible method of long-segment reconstruction is devised [5]. Artificial materials do not grow with the child, and cadaveric grafts are often only temporizing [18]. Current research efforts are focused on neotrachea creation via tissue engineering [19]. However, establishing a patent airway may only be the first step in changing the poor prognosis for TA patients. Normal lung size is reported at autopsy [20], but the use of tracheal occlusion in congenital diaphragmatic hernia has demonstrated that the normal lung size is still accompanied by surfactant deficiency [21], increased thickness of the adventitia, and pulmonary hypertension [22]. Like congenital diaphragmatic hernia, the lung function may be problematic even when the primary defect is repaired.

## 4. Conclusion

TA with distal TEF is rare and difficult to diagnose prenatally. TA should be considered in neonates with apnea, absence of crying, inability to intubate, and acute decompensation triggered by gastric decompression. LMA may be successfully used to stabilize and transport the patient until a definitive diagnosis can be made.

## Data Availability

No data were used to support this study.

## Supplementary Materials

Supplementary Materials Supplementary Video: rigid laryngoscopy of vocal cords and subglottis: the petechiae present on the distal wall of the blind-ended laryngeal pouch from previous intubation attempts are noted. No tracheal rings are present.
